# Quality of life in patients with schizophrenia: A 2-year cohort study in primary mental health care in rural China

**DOI:** 10.3389/fpubh.2022.983733

**Published:** 2022-09-07

**Authors:** Xiao-Yan He, Christine Migliorini, Zhuo-Hui Huang, Fei Wang, Rui Zhou, Zi-Lang Chen, Yao-Nan Xiao, Qian-Wen Wang, Shi-Bin Wang, Carol Harvey, Cai-Lan Hou

**Affiliations:** ^1^Liuzhou Worker's Hospital, Liuzhou, China; ^2^Guangdong Provincial People's Hospital, Guangdong Academy of Medical Sciences, Guangdong Mental Health Center, Guangzhou, China; ^3^Psychosocial Research Centre, Department of Psychiatry, University of Melbourne, Melbourne, VIC, Australia; ^4^Luoding Mental Health Center, Yunfu, China; ^5^Peking University Institute of Mental Health, NHC Key Laboratory of Mental Health (Peking University), National Clinical Research Center for Mental Disorders (Peking University Sixth Hospital), Beijing, China

**Keywords:** schizophrenia, quality of life, primary care, risk factors, China

## Abstract

**Objective:**

Quality of life (QoL) has been always an important way to evaluate the outcomes of schizophrenia, but there have been few previous longitudinal studies and few in middle-income countries. This study aimed to explore the QoL in Chinese patients with schizophrenia treated in primary mental health care and the risk factors of QoL over time.

**Methods:**

Patients with schizophrenia treated in primary mental health care in rural/regional areas in Luoding, Guangdong, PR China, were evaluated with an extended questionnaire including the Chinese version of the World Health Organization Quality of Life (WHOQOL-BREF) at baseline and 2-year follow-up. Bivariate and multivariate analyses were conducted including Generalized Estimated Equation analyses (GEE).

**Results:**

Four hundred and ninety-one patients with schizophrenia in primary care completed the 2-year follow up evaluation. The QoL physical, environmental, and social relationships domains showed improvement after the 2-year period, but the psychological domain did not. GEE results showed that earlier age of onset, older age, being employed, being unmarried, the thicker waist circumference, less use of clozapine or other SGAs, fewer hospitalizations, more frequent insomnia, more severe depressive and negative symptoms as well as worse treatment insight were independently associated with poor QoL in patients with schizophrenia.

**Conclusion:**

According to our results, to improve the quality of life of patients with schizophrenia in primary care, we should pay more attention to the treatment of depression, negative and insomnia symptoms of schizophrenia, the choice and dosage of antipsychotic medication and improvement in the treatment compliance. The combined use of educational and behavioral strategies may improve treatment adherence.

## Introduction

Schizophrenia, one of the most common severe mental diseases, seriously undermines human health and is associated with a heavy socio-economic burden on patients' families and society (1). Traditionally, schizophrenia research has majorly focused on symptoms, clinical outcomes, morbidity, treatment modalities, and prediction of clinical syndrome recovery (2, 3). However, to provide comprehensive services to patients with schizophrenia and better support their return to community life, studies have increasingly paid attention to their quality of life (QoL).

WHO defines Quality of Life as an individual's perception of their position in life in the context of the culture and value systems in which they live and in relation to their goals, expectations, standards and concerns (Available from https://www.who.int/tools/whoqol). QoL has become an important outcome for evaluating the treatment outcomes of schizophrenia (4, 5). Boyer et al. reported that QoL is one of the most important features for predicting the recurrence of schizophrenia. The higher the patient's QoL, the lower the likelihood of relapse within 2 years (6).

Compared to healthy controls or their non-affected siblings, the QoL for patients with schizophrenia has been shown to be markedly reduced (7, 8). A large number of studies, including meta-analyses, have evaluated the QoL and its influential factors in schizophrenic patients (9). Severe positive and negative psychotic symptoms (10), severe depressive symptoms (11, 12), and worse sleeping disturbance patterns (13) are associated with worse quality of life as well as low levels of social functioning. Antipsychotics are extremely important for treatment of schizophrenia. In the past 60 years, various studies have published on the use of antipsychotics for treatment of schizophrenia, with the major research trends being: (1) antipsychotic efficacy; (2) cognition in schizophrenia; (3) side effects of antipsychotics (14), all of which affect the QoL of schizophrenia patients.

However, most of these studies were based on cross-sectional designs. Longitudinal, repeated measures designs using large study samples are better placed to inform on factors that can influence QoL in people with schizophrenia.

Further, most studies were performed in western countries. The QoL is influenced by socio-cultural and economic factors (15, 16). Xiang et al. showed that severe positive symptoms are predictive of low levels of social and environmental domains of QoL in Chinese patients. This finding is contrary to the commonly held belief that QoL is highly associated with negative symptoms and weakly associated or independent of positive symptoms (17).

Compared to developed countries (18, 19), China's communal mental health system is not well-established. QoL varies between countries and is correlated with national economic growth, spiritual budget, and other factors (20). Therefore, we adopted a 2-year longitudinal design to assess the relative effects of demographic and clinical characteristics to the QoL for patients with schizophrenia in the community and managed in a primary mental health system in a non-western country.

## Study participants and methods

### Ethical considerations

This study was jointly undertaken by the Guangdong mental health center, Guangdong Province, China, and the University of Melbourne, with ethical approval obtained at both institutions. All participants, as well as their guardians, gave written informed consents.

### Study participants

Schizophrenic patients were selected from the China National Psychiatric Management System (CNPMS) through a random cluster sampling method. CNPMS is a Chinese system for registration and management of patients with severe mental illnesses, including schizophrenia, bipolar disorders, paranoid psychosis, schizoaffective disorders, mental disorders related to epilepsy, and intellectual disability with psychotic symptoms. In this study, all patients were from Luoding city, an underdeveloped rural area in Guangdong Province.

The inclusion criteria were: (a) Aged above 18; (b) Have an ICD-10 diagnosis of schizophrenia, not including ultra-high risk for psychosis (UHR), but included first-episode schizophrenia (FEP), as it was not specifically distinguished between patients with first-episode or recurrent schizophrenia; (c) Living in Luoding city and receiving treatment in outpatient clinics or in the community; (d) Able to understand and read questionnaire contents.

**Figure 1:**
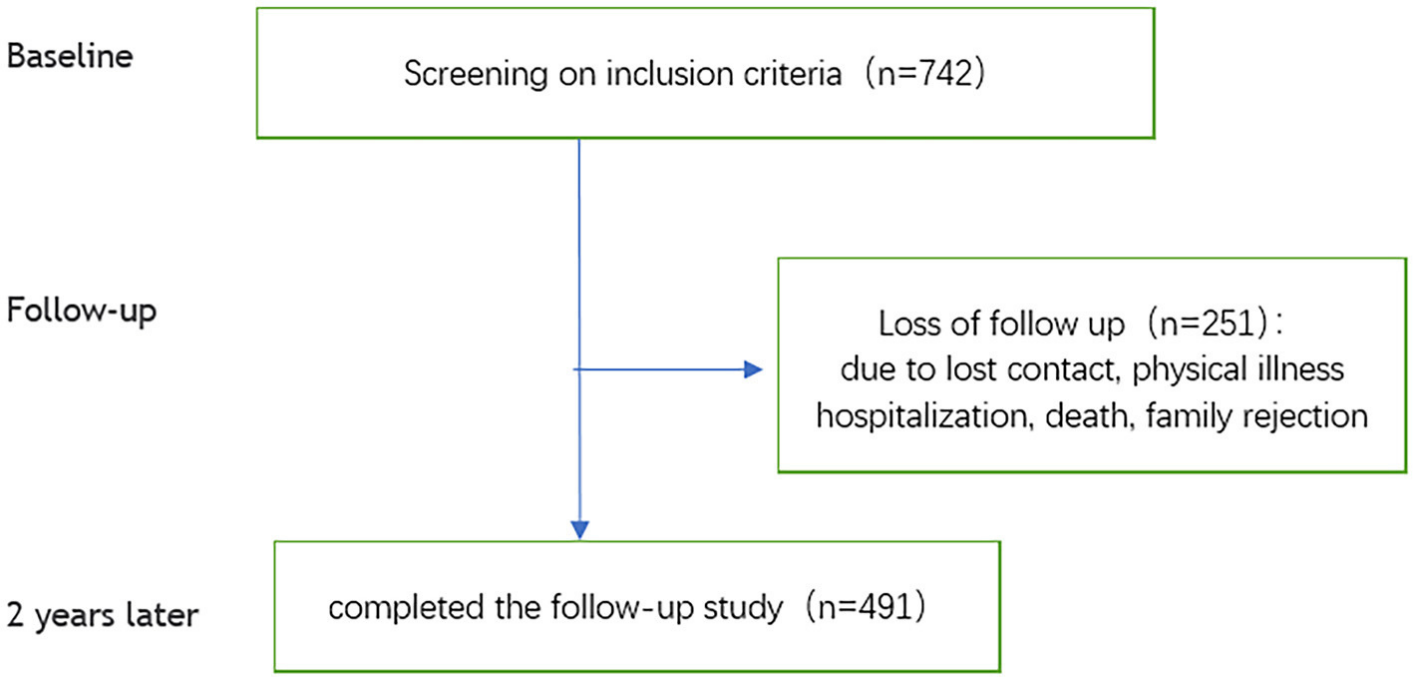
Flowchart of the study participants.

First, the sampled schizophrenic patients were contacted by telephone to clarify the details of the research protocol. If they were willing to participate, one of three psychiatrists, each with more than 5 years of clinical experience, conducted a face-to-face interview with patients. After the interview, patients were included in the study if they met the inclusion criteria. A detailed description of baseline characteristics and data collection is provided elsewhere (21). Two years later, we followed up on enrolled individuals. The flowchart for this study is shown in Figure 1.

### Assessments

Data on socio-demographic variables (age, gender, marital status, education level, employment status) and clinical characteristics [number of hospitalizations, first episode or not, age of onset of illness, Body Mass Index (BMI)] were collected at interview. Data on the use of first-generation antipsychotics (FGAs), second-generation antipsychotics (SGAs), antidepressants, mood stabilizers, benzodiazepines, and anticholinergic drugs were obtained from medical records.

Symptomatology was determined using the Chinese version of the Brief Psychiatric Rating Scale (BPRS), which had been tested for reliability and validity (22, 23). The BPRS was used to rate the presence or absence of psychiatric symptoms, and the severity of each symptom; it consists of 18 items and each item is graded from 1 to 7 (where a rating of seven indicates extreme severe). The higher the score, the more severe the symptom.

Depressive symptoms were assessed using the Chinese version of the Montgomery-Åsberg Depression Rating Scale (MADRS), with good reliability and validity (24, 25). The scale has 10 items assessed on a 7-point scale. Typically, a score of <12 indicates no depressive symptoms, with higher scores representing greater depressive symptoms.

Insight was measured using the Chinese version of the Insight and Treatment Attitude Questionnaire (ITAQ), which had been tested for reliability and validity (26, 27). The ITAQ scale includes 11 items (five items: awareness into illness or illness insight, six items: awareness into need for medical/hospitalization treatment or treatment insight). Items are rated on a scale of 0 (no insight) to 2 (good insight), and a higher total score means better insight.

An eight-question self-report scale was used to evaluate the sleep status of participants over the past month (8, 28). The scale included four sub-domains: any type of insomnia [e.g., difficulty initiating sleep (Over the past month, have you had trouble falling asleep), difficulty maintaining sleep (Over the past month, have you had difficulty maintaining sleep for a long time or waking up from time to time?) and early morning wakening (For the past month, have you had trouble waking up in the middle of the night or waking up too early to go to sleep again)]; whether insomnia requires treatment [Have you been using sleeping pills (medicines) for insomnia for the past 1 month?]; whether bothered by insomnia [Have you been bothered by insomnia for the past 1 month?]; whether insomnia affects life, work and study [Have you suffered from insomnia that affects life, work, and study for the past 1 month?].

We used a six-question self-report scale to assess suicide-related problems of participants over the past 2 weeks. The scale has three domains: whether patients had suicidal thoughts, suicidal behaviors or had attempted suicide (29). Suicidality was judged to have occurred in the past 2 weeks if one or more of the three aspects were answered in the affirmative.

Quality of life was measured using the Chinese version of the abridged World Health Organization Quality of Life instrument (WHOQOL-BREF), which had been tested for reliability and validity (30). The WHOQOL-BREF scale consists of 28 items, in which each item is scored on a 5-point Likert-scale that measures four domains: physical health (seven-items), psychological health (six-items), social relationship (three-items), environmental health (eight-items) plus four items. Only the four domain scores were used in analyses. A higher score reflects better quality of life.

Before study implementation, three investigators performed the inter-rater reliability training for 20 schizophrenic patients. Inter-rater reliability training of the tools yielded satisfactory agreement (>0.90).

### Statistical analysis

The IBM SPSS software version 22.0 and 25.0 was used for analyses. A total of 491 patients completed the follow-up. Socio-demographic and clinical characteristics of schizophrenic patients who completed the 2 years of follow-up and those who were lost to follow-up were compared using the independent samples *t*-test and Chi square tests. We analyzed differences in socio-demographic, clinical characteristics, and QoL domains between baseline and follow-up groups using the paired samples *t*-test and McNemar test. QoL was compared between patients with schizophrenia (at baseline, after 2 years) and the general Chinese population (NORM) using the summary independent samples *t*-test. In large scale studies, very small effects may reach statistical significance. To determine whether effects have a relevant magnitude, effect sizes are used to describe the strength of a phenomenon. Conventionally, when comparing two mean values, the Cohen *d* value is calculated as the effect size, where 0.2 represents a small effect size, 0.5 represents a medium effect size, and 0.8 represents a large effect size (31). When comparing dichotomous variables, the *phi* value is calculated as the effect size, whereby 0.1 represents a small effect size, 0.3 represents a medium effect size, and 0.5 represents a large effect size (32).

Generalized Estimating Equations (GEE) were used to assess the risk factors for QoL domains in the cohort of 491 patients with schizophrenia. GEE is mainly used for analysis of repeated measurement data, such as longitudinal observation of epidemiological populations, where there are more flexible observation time points. For predictor variables, time dependent variables can be included. Therefore, GEE is suitable for statistical analysis in this study. The selection of independent variables in GEE analysis was based on clinical experience, other relevant studies and on positive results. The dependent variable was the follow-up WHOQOL-BREF score. *p* ≤ 0.05 was the threshold for statistical significance (two-tailed).

## Results

### Sample characteristics and descriptive analyses

A total of 742 patients completed the first assessment, accounting for about 30% of all patients diagnosed with schizophrenia and registered by CNPMS in Luoding City. Four hundred and ninety-one participants (66% of the baseline sample) completed both baseline and follow-up measures. Analyses of socio-demographic and clinical characteristics of patients who completed follow-up (*n* = 491) and those who were lost to follow-up (*n* = 251) showed that participants who were lost to follow-up were more likely to be living with others (*p* = 0.049, *phi* = 0.07), had fewer number of hospitalizations over their lifetime (*p* = 0.02, *d* = 0.16), were more likely to be current smokers (*p* = 0.02, *phi* = 0.08), and were less likely to be prescribed with clozapine (*p* = 0.003, *phi* = 0.11). However, the effect sizes were small to very small in each instance (Table 1).

**Table 1:** Sociodemographic and clinical characteristics of the study sample.

	**Completed follow-up**		**Loss to follow-up**		**Statistics**		**Effect size**	
	**sample (**= **491)**		**sample (*****n*** = **251)**	
	***n***	***%***	***n***	***%***	**χ^2a^**	***df***	***p*-Value**	***Phi***
Male	313	63.74	149	59.36	1.36	1	0.24	0.04
Married	213	43.38	115	45.82	0.40	1	0.52	−0.02
Employed	318	64.76	170	67.73	0.65	1	0.42	−0.03
Living with others	453	92.26	241	96.02	3.87	1	**0.05**	−0.07
**Current drinker**	**20**	**4.1**	**8**	**3.2**	**0.3**	**1**	**0.54**	**0.02**
**Current smoker**	**122**	**24.8**	**44**	**17.5**	**5.1**	**1**	**0.02**	**0.08**
First episode	47	9.57	33	13.15	2.21	1	0.14	−0.06
Physical disease	27	5.50	22	8.76	2.87	1	0.09	0.06
Family psychiatric history	111	22.61	57	22.71	0.001	1	0.98	−0.001
On clozapine	104	21.18	31	12.35	8.70	1	**0.003**	0.11
On FGAs	143	29.12	57	22.71	3.47	1	0.06	0.07
On SGAs other than clozapine	226	46.03	120	47.81	0.21	1	0.65	−0.02
On antidepressant	8	1.63	4	1.59	0.001	1	0.97	0.001
On benzodiazepine	22	4.48	11	4.38	0.004	1	0.95	0.002
On mood stabilizer	58	11.81	23	9.16	1.20	1	0.27	0.04
On anticholinergic	164	33.40	81	32.27	0.09	1	0.75	0.01
Insomnia in past 1 month	241	49.08	121	48.21	0.05	1	0.82	0.01
Insomnia treatment	71	14.46	37	14.74	0.01	1	0.92	−0.004
Insomnia bother	176	35.85	84	33.47	0.41	1	0.52	0.02
Insomnia affects life	167	34.01	87	34.66	0.03	1	0.86	−0.006
Suicide related issues in past 2 weeks	5	1.02	2	0.79	0.09	1	0.76	0.01
	**Mean**	***SD***	**Mean**	***SD***	***t* ^b^**	***df***	***p*** **-Value**	**Cohen's** ***d***
Age (years)	40.35	11.91	38.86	13.42	1.48	740	0.12	0.12
Education level (years)	8.27	2.16	8.17	2.30	0.56	740	0.58	0.05
Age of onset (years)	26.20	9.18	25.79	9.24	0.58	740	0.56	0.05
Number of hospitalizations	2.16	1.92	1.80	1.97	2.34	740	**0.02**	0.16
Waist circumference	64.25	7.89	63.88	8.36	0.76	740	0.45	0.06
BMI (kg/m^2^)	21.73	2.48	21.71	2.56	0.22	740	0.83	0
BPRS total	25.89	8.26	26.34	8.30	−0.70	740	0.48	−0.06
BPRS positive	6.12	2.73	6.08	2.56	0.20	740	0.85	0.01
BPRS negative	5.54	2.77	5.50	2.91	0.20	740	0.84	0.01
BPRS affect	5.13	1.73	5.08	1.57	0.42	740	0.67	−0.10
MADRS total	4.69	5.70	5.29	5.97	−1.34	740	0.18	0.03
ITAQ total	9.83	7.78	9.68	7.73	0.24	740	0.81	0.03
ITAQ illness	3.97	3.39	3.84	3.43	0.47	740	0.63	0.03
ITAQ medication	5.86	4.60	5.84	4.54	0.06	740	0.95	0
WHOQOL-BREF physical health	12.26	1.41	12.10	1.53	1.48	740	0.14	0.07
WHOQOL-BREF psychological health	12.53	1.22	12.44	1.40	0.88	740	0.38	0.08
WHOQOL-BREF social relationships	12.10	2.09	11.81	1.87	1.83	740	0.07	0.15
WHOQOL-BREF environment	11.87	1.29	11.84	1.38	0.22	740	0.82	0

Bold values: p < 0.05.

^a^Pearson chi-square test.

^b^Independent-Samples T-Test.

BMI, Body Mass Index; BPRS, Brief Psychiatric Rating Scale; FGAs, First-generation antipsychotics; ITAQ, Insight and Treatment Attitude Questionnaire; MADRS, Montgomery–Asberg Depression Rating Scale; SGAs, second-generation antipsychotics; SDS, Sheehan Disability Scale; WHOQOL-BREF, The World Health Organization Quality of Life- brief version.

### Changes in demographic and clinical variables

Bivariate comparison analyses revealed that at follow-up, participants were more likely to be unemployed (medium effect size). The number of medications prescribed increased over the 2-year period, with significant increases noted for all types, except FGAs (Table 2). Over the 2-year period, the BMI markedly increased, as did waist circumference.

**Table 2:** Comparison of sociodemographic and clinical characteristics between baseline and 2 year follow-up in 491 patients with schizophrenia.

	**Baseline**		**Follow-up**		**Statistics**		**Effect size**	
	***n***	***%***	***n***	***%***	**χ^2a^**	***df***	***p*-Value**	***Phi***
Married	213	43.38	220	44.81	0.43	1	0.510	0.657
Employed	318	64.76	190	38.69	70.13	1	**<0.001**	0.140
Physical violence in past 1 year	205	41.75	198	40.32	0.17	1	0.677	0.129
On FGAs	143	29.12	162	32.99	3.02	1	0.081	0.494
On clozapine	104	21.18	164	33.40	29.50	1	**<0.001**	0.454
On SGAs other than clozapine	153	31.16	288	58.66	46.64	1	**<0.001**	−0.551
On antidepressant	8	1.63	19	3.87	13.47	1	**<0.001**	0.482
On benzodiazepine	22	4.48	75	15.27	34.23	1	**<0.001**	0.154
On mood stabilizer	58	11.81	109	22.20	22.12	1	**<0.001**	0.214
On anticholinergic	164	33.40	203	41.34	7.89	1	**0.005**	0.212
Insomnia in past 1 month	241	49.08	232	47.25	0.28	1	0.595	0.074
Insomnia treatment	71	14.46	81	16.50	0.66	1	0.415	0.051
Insomnia bother	176	35.85	87	17.72	42.79	1	**<0.001**	0.109
Insomnia affects life	167	34.01	65	13.24	59.31	1	**<0.001**	0.100
suicide related issues in 2 weeks	5	1.02	17	3.46	6.72	1	**0.008**	0.203
	**Mean**	***SD***	**Mean**	***SD***	***t* ^b^**	***df***	***p*** **-Value**	**Cohen's** ***d***
Number of hospitalizations	2.16	1.92	3.06	2.58	−16.23	490	**<0.001**	0.732
Waist circumference	64.25	7.89	88.95	11.89	−38.77	471	**<0.001**	1.784
BMI (kg/m^2^)	21.73	2.48	24.21	4.13	−11.27	473	**<0.001**	0.518
BPRS total	25.89	8.26	24.96	6.38	2.16	490	**0.032**	0.0.97
BPRS positive	6.12	2.73	5.85	2.39	1.87	490	0.062	0.084
BPRS negative	5.54	2.77	5.00	2.76	4.00	490	**<0.001**	0.181
BPRS affect	5.13	1.73	5.54	1.68	−3.94	490	**<0.001**	0.178
MADRS total	4.69	5.70	5.77	6.02	−3.05	490	**0.002**	0.137
ITAQ total	9.83	7.78	9.47	6.39	0.95	490	0.342	0.043
ITAQ illness	3.97	3.39	3.43	2.82	3.29	490	**0.001**	0.148
ITAQ medication	5.86	4.60	6.04	3.92	−0.81	490	0.416	0.037
WHOQOL-BREF physical health	12.26	1.41	13.92	1.73	−17.60	490	**<0.001**	0.794
WHOQOL-BREF psychological health	12.53	1.22	12.57	1.98	−0.43	490	0.668	0.019
WHOQOL-BREF social relationships	12.10	2.09	12.78	2.38	−4.88	490	**<0.001**	0.220
WHOQOL-BREF environment	11.87	1.29	12.42	1.77	−5.88	490	**<0.001**	0.265

Bold values: p < 0.05.

^a^McNemar Test.

^b^Paired-samples T-Test.

BMI, Body Mass Index; BPRS, Brief Psychiatric Rating Scale; FGAs, First-generation antipsychotics; ITAQ, Insight and Treatment Attitude Questionnaire; MADRS, Montgomery–Asberg Depression Rating Scale; SGAs, second-generation antipsychotics; SDS, Sheehan Disability Scale; WHOQOL-BREF, The World Health Organization Quality of Life- brief version.

While the number of participants having, and receiving treatment for insomnia was relatively stable, fewer participants reported being bothered by insomnia or insomnia affecting their lives. At follow-up, significantly more participants reported having suicide-related thoughts, behaviors or attempts in previous 2 weeks (Table 2).

At 2-year follow up, there were significant increases in depression symptoms (*p* = 0.002, *d* = 0.137), which affected the symptomatic score (*p* < 0.001, *d* = 0.178), while there were decreases (i.e., improvements) in BPRS total (*p* = 0.032, *d* = 0.097) and negative symptom scores (*p* < 0.001, *d* = 0.181). Although changes in positive symptoms were not significant, there was a trend toward improvement (*p* = 0.062, *d* = 0.084). In contrast, there was a decrease (i.e., worsening) in ITAQ illness scores (*p* = 0.001, *d* = 0.148). After 2 years, there was a general improvement in other domains of QoL, besides the psychological domain.

Also, the results showed that all four aspects of QoL in schizophrenia patients were poorer at baseline relative to the Chinese general population, and the QoL after 2 years was still poorer than that of the Chinese general population except for the environmental domain, which was higher than that of the Chinese general population (Table 3).

**Table 3:** Comparison of QoL between schizophrenia patients (at baseline, after 2 years) and the general Chinese population.

	①		②		③		**Statistics**①③***^a^***		**Statistics**②③	
	**Mean**	***SD***	**Mean**	***SD***	**Mean**	***SD***	***t***	***p*-Value**	***t***	***p*-Value**
WHOQOL-BREF physical health	12.21	1.46	13.92	1.73	15.10	2.30	29.08	**<0.001**	9.76	**<0.001**
WHOQOL-BREF psychological health	12.50	1.28	12.57	1.98	13.89	1.89	16.71	**<0.001**	11.89	**<0.001**
WHOQOL-BREF social relationships	12.00	2.02	12.78	2.38	13.93	2.06	18.43	**<0.001**	9.11	**<0.001**
WHOQOL-BREF environment	11.86	1.32	12.42	1.77	12.14	2.08	3.12	**0.002**	2.56	**0.01**

Bold values: p < 0.05.

^a^Summary independent samples T-Test.

baseline (n = 742); follow-up (n = 491); General population pattern in China (n = 777).

WHOQOL-BREF, The World Health Organization Quality of Life- brief version.

### Risk factors of WHOQOL domains (GEE)

Generalized Estimated Equation analyses revealed that early onset age, being employed, reduced use of SGAs other than clozapine, fewer lifetime hospitalizations, frequent insomnia as well as more severe depressive and negative symptoms were independently associated with poor QoL physical domain (Table 4), while early onset age, less use of clozapine, fewer lifetime hospitalizations, thicker waist circumference, more frequent insomnia, as well as more severe depressive and negative symptoms were independently associated with poor QoL psychological domain (Table 5). Moreover, early onset age, being unmarried, older age, less use of SGAs except clozapine, fewer lifetime hospitalizations, more frequent insomnia, more severe depressive and negative symptoms, as well as worse treatment insights were independently associated with poor QoL social relationship domain (Table 6). Early onset age, less use of SGAs except clozapine, fewer lifetime hospitalizations, more frequent insomnia, more severe depressive and negative symptoms, as well as worse treatment insights were independently associated with poor QoL environment domain (Table 7). To illustrate these results, a summary table was made regarding the four aspects of QoL (Table 8).

**Table 4:** Generalized Estimating Equations (GEE) results modeling risk factors associated with WHOQOL physical domain in 491 patients with schizophrenia.

**Baseline variables^a^**	***Coef***	***SE***	***p*-Values**	***OR***	***95% CI(OR)***
Male	0.06	0.12	0.63	1.06	0.82~1.36
Living with others	0.15	0.18	0.41	1.16	0.808~1.67
First episode	−0.04	0.17	0.809	0.95	0.68~1.34
Current drinker	−0.07	0.24	0.77	0.93	0.57~1.51
Current smoker	0.15	0.11	0.19	1.16	0.92~1.47
Physical disease	0.04	0.22	0.85	1.04	0.66~1.62
Family psychiatric history	−0.109	0.12	0.37	0.89	0.703~1.14
Physical violence in past 1 year	−0.17	0.109	0.11	0.84	0.67~1.04
Education level (years)	0.01	0.02	0.58	1.01	0.96~1.06
Age of onset (years)	0.01	0.008	**0.04**	1.01	1.00~1.03
**Time-dependent variables** ^**b**^
Married	0.03	0.13	0.77	1.03	0.801~1.34
Employed	−0.39	0.109	**<0.001**	0.67	0.54~0.83
On FGAs	0.02	0.12	0.82	1.02	0.805~1.31
On Clozapine	0.19	0.12	0.105	1.21	0.96~1.53
On SGAs other than clozapine	0.38	0.11	**0.001**	1.46	1.15~1.84
On antidepressant	−0.48	0.35	0.16	0.61	0.308~1.22
On benzodiazepine	0.24	0.21	0.24	1.27	0.84~1.92
On mood stabilizer	−0.12	0.14	0.37	0.88	0.66~1.16
On Anticholinergic	−0.15	0.11	0.18	0.85	0.67~1.07
insomnia in past 1 month	−0.49	0.108	**<0.001**	0.61	0.49~0.75
Suicide related issues in 2 weeks	0.51	0.37	0.16	1.66	0.804~3.46
Age (years)	−0.002	0.006	0.72	0.99	0.98~1.01
Number of hospitalizations	0.07	0.02	**0.01**	1.07	1.01~1.13
Waist circumference	<0.001	<0.001	0.73	1.00	0.99~1.002
BMI (kg/m^2^)	<0.001	<0.001	0.73	1.00	0.99~1.002
BPRS positive	0.009	0.03	0.78	1.009	0.94~1.07
BPRS negative	−0.12	0.02	**<0.001**	0.88	0.83~0.92
BPRS affect	−0.03	0.03	0.29	0.96	0.89~1.03
MADRS total	−0.03	0.01	**0.002**	0.96	0.94~0.98
ITAQ illness	−0.05	0.03	0.11	0.95	0.89~1.01
ITAQ medication	0.03	0.02	0.18	1.03	0.98~1.08

Bold values: p < 0.05.

^a^The value of baseline variables would not change during the whole follow-up period.

^b^The value of time-dependent variables would change during follow-up period.

BPRS, Brief Psychiatric Rating Scale; FGAs, First-generation antipsychotics; ITAQ, Insight and Treatment Attitude Questionnaire; MADRS, Montgomery–Asberg Depression Rating Scale; SGAs, second-generation antipsychotics.

**Table 5:** Generalized Estimating Equations (GEE) results modeling risk factors associated with WHOQOL psychological domain in 491 patients with schizophrenia.

**Baseline variables^a^**	***Coef***	***SE***	***p*-Value**	***OR***	**95*% CI (OR)***
Male	0.17	0.11	0.12	1.19	0.95~1.48
Living with others	−0.06	0.17	0.702	0.93	0.66~1.31
First episode	0.22	0.16	0.15	1.25	0.91~1.72
Current drinker	−0.12	0.24	0.607	0.88	0.54~1.43
Current smoker	0.06	0.11	0.59	1.06	0.84~1.34
Physical disease	−0.303	0.21	0.15	0.73	0.48~1.11
Family psychiatric history	−0.007	0.11	0.95	0.99	0.79~1.24
Physical violence in past 1 year	−0.17	0.10	0.08	0.84	0.69~1.02
Education level (years)	0.03	0.02	0.14	1.03	0.98~1.08
Age of onset (years)	0.03	0.007	**<0.001**	1.03	1.02~1.04
**Time-dependent variables** ^**b**^
Married	−0.16	0.11	0.17	0.85	0.67~1.07
Employed	0.17	0.09	0.08	1.18	0.97~1.44
On FGAs	−0.07	0.105	0.508	0.93	0.75~1.14
On clozapine	0.29	0.12	**0.01**	1.33	1.05~1.69
On SGAs other than clozapine	0.17	0.102	0.08	1.19	0.97~1.45
On antidepressant	−0.11	0.29	0.69	0.88	0.49~1.59
On benzodiazepine	−0.01	0.17	0.91	0.98	0.69~1.38
On mood stabilizer	−0.07	0.14	0.61	0.93	0.705~1.22
On anticholinergic	−0.17	0.108	0.106	0.84	0.67~1.03
Insomnia in past 1 month	−0.33	0.09	**0.001**	0.71	0.58~0.86
Suicide related issues in past 2 weeks	−0.21	0.43	0.62	0.81	0.34~1.88
Age (years)	−0.01	0.005	**0.01**	0.98	0.97~0.99
Number of hospitalizations	0.08	0.02	**<0.001**	1.09	1.04~1.14
Waist circumference	−0.002	<0.001	**0.04**	0.99	0.99~1.00
BMI (kg/m^2^)	0.001	<0.001	0.13	1.001	1.00~1.003
BPRS positive	0.04	0.02	0.08	1.04	0.99~1.103
BPRS negative	−0.07	0.02	**0.002**	0.92	0.88~0.97
BPRS affect	−0.01	0.03	0.72	0.98	0.92~1.05
MADRS total	−0.06	0.01	**<0.001**	0.94	0.92~0.96
ITAQ Illness	−0.02	0.02	0.43	0.97	0.92~1.03
ITAQ Medication	0.05	0.02	0.02	1.05	1.005~1.10

Bold values: p < 0.05.

^a^The value of baseline variables would not change during the whole follow-up period.

^b^The value of time-dependent variables would change during follow-up period.

BPRS, Brief Psychiatric Rating Scale; FGAs, First-generation antipsychotics; ITAQ, Insight and Treatment Attitude Questionnaire; MADRS, Montgomery–Asberg Depression Rating Scale; SGAs, second-generation antipsychotics.

**Table 6:** Generalized Estimating Equations (GEE) results modeling risk factors associated with WHOQOL Social Relationship domain in 491 patients with schizophrenia.

**Baseline variables^a^**	***Coef***	***SE***	***p*-Value**	***OR***	**95*%CI (OR)***
Male	0.01	0.16	0.93	1.01	0.73~1.39
Living with others	0.16	0.26	0.53	1.18	0.701~1.99
First episode	−0.15	0.23	0.51	0.85	0.54~1.35
Current drinker	−0.17	0.36	0.62	0.83	0.406~1.72
Current smoker	−0.04	0.17	0.82	0.96	0.67~1.36
Physical disease	0.02	0.29	0.93	1.02	0.57~1.82
Family psychiatric history	0.01	0.15	0.903	1.02	0.74~1.39
Physical violence in past 1 year	−0.03	0.14	0.803	0.96	0.72~1.28
Education level (years)	0.04	0.03	0.12	1.04	0.98~1.11
Age of onset (years)	0.03	0.01	**<0.001**	1.03	1.01~1.06
**Time-dependent variables** ^**b**^
Married	0.63	0.17	**<0.001**	1.88	1.35~2.63
Employed	−0.104	0.13	0.45	0.901	0.68~1.18
On FGAs	−0.20	0.16	0.21	0.81	0.59~1.12
On clozapine	−0.14	0.15	0.34	0.86	0.64~1.16
On SGAs other than clozapine	0.33	0.15	**0.03**	1.39	1.02~1.88
On antidepressant	−0.28	0.36	0.44	0.75	0.36~1.54
On benzodiazepine	0.23	0.25	0.36	1.25	0.76~2.06
On mood stabilizer	−0.23	0.18	0.201	0.79	0.55~1.13
On anticholinergic	−0.204	0.15	0.19	0.81	0.60~1.11
Insomnia in past 1 month	−0.49	0.14	**<0.001**	0.607	0.45~0.803
Suicide related issues in past 2 weeks	−0.21	0.49	0.65	0.805	0.307~2.11
Age (years)	−0.03	0.008	**<0.001**	0.97	0.95~0.98
Number of hospitalizations	0.07	0.03	**0.02**	1.07	1.01~1.14
Waist circumference	−0.001	<0.001	0.19	0.99	0.99~1.001
BMI (kg/m^2^)	0.001	0.001	0.407	1.001	0.99~1.003
BPRS positive	−0.008	0.03	0.82	0.99	0.92~1.06
BPRS negative	−0.14	0.03	**<0.001**	0.86	0.81~0.92
BPRS affect	−0.07	0.04	0.14	0.93	0.84~1.02
MADRS total	−0.03	0.01	**0.02**	0.96	0.93~0.99
ITAQ illness	−0.07	0.03	0.07	0.93	0.86~1.007
ITAQ medication	0.07	0.02	**0.01**	1.07	1.01~1.13

Bold values: p < 0.05.

^a^The value of baseline variables would not change during the whole follow-up period; ^b^The value of time-dependent variables would change during follow-up period.

BPRS, Brief Psychiatric Rating Scale; FGAs, first-generation antipsychotics; ITAQ, Insight and Treatment Attitude Questionnaire; MADRS, Montgomery–Asberg Depression Rating Scale; SGAs, second-generation antipsychotics.

**Table 7:** Generalized Estimating Equations (GEE) results modeling risk factors associated with WHOQOL environment domain in 491 patients with schizophrenia.

**Baseline variables^a^**	***Coef***	***SE***	***p*-Value**	***OR***	**95*%CI(OR)***
Male	0.07	0.11	0.49	1.08	0.86~1.35
Living with others	0.22	0.17	0.19	1.25	0.89~1.75
First episode	0.07	0.16	0.62	1.08	0.79~1.48
Current drinker	−0.45	0.28	0.102	0.63	0.36~1.09
Current smoker	0.009	0.11	0.93	1.009	0.809~1.25
Physical disease	−0.25	0.202	0.21	0.77	0.52~1.15
Family psychiatric history	0.09	0.11	0.41	1.09	0.87~1.37
Physical violence in past 1 year	−0.16	0.102	0.11	0.85	0.69~1.04
Education level (years)	0.04	0.02	0.06	1.04	0.99~1.08
Age of onset (years)	0.02	0.007	**0.003**	1.02	1.007~1.03
**Time-dependent variables** ^**b**^
Married	0.07	0.11	0.48	1.08	0.86~1.34
Employed	−0.17	0.09	0.07	0.84	0.69~1.01
On FGAs	−0.01	0.101	0.87	0.98	0.807~1.20
On clozapine	0.28	0.11	**0.01**	1.32	1.06~1.64
On SGAs other than clozapine	0.24	0.102	**0.01**	1.27	1.04~1.56
On antidepressant	−0.201	0.28	0.48	0.81	0.46~1.44
On benzodiazepine	−0.03	0.18	0.85	0.96	0.67~1.38
On mood stabilizer	−0.17	0.13	0.21	0.84	0.64~1.105
On Anticholinergic	−0.04	0.09	0.69	0.96	0.79~1.16
Insomnia in past 1 month	−0.33	0.09	**0.001**	0.71	0.58~0.87
Suicide related issues in past 2 weeks	−0.05	0.38	0.88	0.94	0.44~2.01
Age (years)	−0.001	0.005	0.85	0.99	0.98~1.009
Number of hospitalizations	0.05	0.02	**0.009**	1.05	1.01~1.107
Waist circumference	<0.001	<0.001	0.84	1.00	0.99~1.002
BMI (kg/m^2^)	<0.001	<0.001	0.89	1.00	0.99~1.002
BPRS positive	−0.02	0.02	0.39	0.98	0.93~1.02
BPRS negative	−0.08	0.02	**0.001**	0.92	0.87~0.96
BPRS affect	0.006	0.03	0.85	1.006	0.94~1.07
MADRS total	−0.03	0.01	**0.007**	0.97	0.95~0.99
ITAQ illness	−0.04	0.02	0.11	0.95	0.89~1.01
ITAQ medication	0.05	0.02	**0.01**	1.05	1.01~1.10

Bold values: p < 0.05.

^a^The value of baseline variables would not change during the whole follow-up period.

^b^The value of time-dependent variables would change during follow-up period.

BPRS, Brief Psychiatric Rating Scale; FGAs, First-generation antipsychotics; ITAQ, Insight and Treatment Attitude Questionnaire; MADRS, Montgomery–Asberg Depression Rating Scale; SGAs, second-generation antipsychotics.

**Table 8:** Summary of generalized estimating equation (GEE) results.

**/**	**WHOQOL**		**WHOQOL**		**WHOQOL social**		**WHOQOL**	
**/**	**physical domain**		**psychological domain**		**relationship domain**		**environment domain**	
	***Coef***	***p*-Value**	***Coef***	***p*-Value**	***Coef***	***p*-Value**	***Coef***	***p*-Value**
Age of onset (years)	0.01	**0.04**	0.03	**<0.001**	0.03	**<0.001**	0.02	**0.003**
Employed	−0.39	**<0.001**	/	**/**	/	**/**	/	**/**
Married	/	**/**	/	**/**	0.63	**<0.001**	/	**/**
On clozapine	/	**/**	0.29	**0.01**	/	**/**	0.28	**0.01**
On SGAs other than clozapine	0.38	**0.001**	/	**/**	0.33	**0.03**	0.24	**0.01**
insomnia in past 1 month	−0.49	**<0.001**	−0.33	**0.001**	−0.49	**<0.001**	−0.33	**0.001**
Age (years)	/	**/**	−0.01	**0.01**	−0.03	**<0.001**	/	**/**
Number of hospitalizations	0.07	**0.01**	0.08	**<0.001**	0.07	**0.02**	0.05	**0.009**
Waist circumference	/	**/**	−0.002	**0.04**	/	**/**	/	**/**
BPRS negative	−0.12	**<0.001**	−0.07	**0.002**	−0.14	**<0.001**	−0.08	**0.001**
MADRS total	−0.03	**0.002**	−0.06	**<0.001**	−0.03	**0.02**	−0.03	**0.007**
ITAQ medication	/	**/**	/	**/**	0.07	**0.01**	0.05	**0.01**

Bold values: p < 0.05.

BPRS, Brief Psychiatric Rating Scale; ITAQ, Insight and Treatment Attitude Questionnaire; MADRS, Montgomery–Asberg Depression Rating Scale; SGAs, second-generation antipsychotics; WHOQOL-BREF, The World Health Organization Quality of Life-brief version.

## Discussion

The results showed that all four aspects of QoL in schizophrenia patients were poorer at baseline relative to the general population, but the QOL after 2 years was still poorer than that of the general population except for the environmental domain, which was higher than that of the general population, probably because the environmental domain mainly examined were living environment, medical insurance, transportation, etc. Probably because of China's development, these aspects improved relatively quickly.

We found that with exception of the psychological domain, there was a general improvement in QoL. The stability of QoL among adults with schizophrenia has yet to be fully established. Some studies reported insignificant changes in QoL for some patients (33), while others reported marked improvements in QoL. These inconsistent outcomes may be due to differences in follow-up times and assessment methods used (34, 35). For instance, Ritsner et al. used the Quality of Life Enjoyment and Life Satisfaction Questionnaire (Q-LES-Q), whereas Gardsjord et al. used the Lehman's Quality of Life Interview to measure subjective quality of life. The mean age at baseline for patients in studies by Ritsner et al. and Gardsjord et al. were 38 and 28 years, respectively. In addition, there are differences in health systems among countries (36). These inconsistent findings warrant further investigations using the same measures across different samples for greater comparability. Regardless of the type of antipsychotic drugs used, patients treated in primary mental health care are usually stable or in remission, which may reduce the impacts of psychopathology as well as side effects on QoL.

Generalized Estimated Equation analysis revealed that the risk factors with significant effects on QoL of patients with schizophrenia included onset age, age, working status, marital status, number of lifetime hospitalizations, treatment with SGAs other than clozapine, treatment with clozapine, insomnia, depressive and negative symptoms.

The earlier the onset age, the worse the QoL for patients living with/diagnosed with schizophrenia, consistent with previous studies (37). It may be that patients with early onset age have poorer educational and vocational adjustment (38), and have worse social functioning (39). This result underscores the importance of early rehabilitation so as to reduce subsequent functional impairment and disability.

We also found that older age has a negative impact on psychological and social relationship domains of the QoL of schizophrenia patients. Consistently, a previous study reported that the QoL in older patients with schizophrenia was lower (8). Similarly, a recent study showed that patients aged 55–64 had lower QoL (40). In addition, a meta-analysis revealed that older age was significantly associated with poorer QoL in schizophrenia patients (41). This may be due to poor general health and higher prevalence of somatic co-morbidity in old age (42).

A higher use of clozapine or other second-generation antipsychotics was significantly associated with improved QoL. It has been reported that second-generation antipsychotics, including clozapine, are able to improve both positive and negative symptoms, while symptom reduction can improve the QoL for schizophrenic patients. In this study, the use of clozapine or other second-generation antipsychotics improved significantly after 2 years, with significant improvements in negative symptoms and insignificant reduction in positive symptoms. Therefore, patients may have improved their QoL through this modality (43, 44).

However, previous studies on the impact of second-generation antipsychotics on QoL have not been conclusively determined. Some studies have argued that compared to first-generation antipsychotics, second-generation antipsychotics fail to significantly improve the QoL of schizophrenic patients due to metabolic syndromes and obesity (45, 46), while some studies found that second-generation antipsychotic drugs can significantly improve the QoL for schizophrenic patients (47, 48). These inconsistent findings may be related to various factors, including the rate of using second-generation antipsychotics, study designs, sample sizes, and selected characteristics among others.

Insomnia is a common sleep disorder in schizophrenic patients (49). In this study, whether at baseline or after 2 years follow-up, about half of the patients suffered from insomnia, which had a significant negative impact on their QoL, in tandem with findings from previous studies (50, 51). Insomnia affects the patient's ability to perform daily tasks and participate in social activities, as well as their ability to function in the society and environment (52–54). This replicated finding is clinically significant, because it points to a window for maintaining the QoL as it provides a basis for clinicians to establish interventions for schizophrenia. Efforts should aim at improving patients' sleep problems using medications or cognitive behavioral therapy, thereby improving patients' QoL (55).

Negative symptoms were also shown to have a negative effect on QoL, consistent with previous studies (56–58). In their meta-analysis, Design et al. showed that culturally-adapted psychosocial interventions for schizophrenia can reduce negative symptoms (59). Therefore, targeting social skills and increasing daily activities for patients with high negative symptoms may be effective for improving the QoL for schizophrenic patients. In addition, physical exercise also had a beneficial for negative symptoms (60).

Depressive symptoms were significantly and negatively associated with each of the QoL domains. This finding is similar to those of previous studies and points to the sweeping deleterious effect of persistent depressive symptoms on QoL of patients with schizophrenia (12, 34). Therefore, the need for active treatment of depression in the full course of treatment of schizophrenia has been reaffirmed (61).

Our study also showed that a better treatment insight rather than illness insight may have a positive impact on both the social relationship and environmental domains of the QoL in patients with schizophrenia (62). Previous studies have shown that the relationship between insight and QoL is related to the different dimensions of insight. Poor insight predicts poorer therapeutic alliance and treatment adherence (63). In China, there are only about 20,000 registered psychiatrists in China serving 1.4 billion people (64). Because of insufficient psychiatric resources, most patients with stable schizophrenia are treated in primary medical institutions. Thus, treatment adherence is particularly important. Increasing the insight into treatment can help patients understand the necessity of following certain treatment methods to recover and the importance of treatment. In addition, careful attention to treatment adjustments over time is warranted, as well as psychosocial interventions, which can improve QoL and combat depression. Studies have shown that antipsychotic treatment can increases patient insight (65).

Unlike previous studies (66–68), this study found that participants hospitalized more often reported better QoL. This may be because the sample used in this study was selected from outpatients or community-based patients in rural areas, and the number of admissions was more indicative of regular care. More communication with physicians and higher adherence may lead to higher QoL.

The impact of marital and working status on QoL suggested the importance of psychosocial and environmental factors for patients with schizophrenia. Our findings showed that being employed had a negative impact on the physical domain of QoL. This result was indeed a little surprising, but may be linked to the observation that only a third of participants were employed after about 2 years. The physical domain was mainly the evaluation of participants' functional-ability. At work, the cognitive decline that participants' experience due to their illness will likely affect their ability to work, increasing the possibility of becoming dissatisfied with themselves, affecting satisfaction in the physical domain. Meanwhile, a previous meta-analysis showed that the causal direction of this association in terms of employment and quality of life remains unclear because studies on the bidirectional relationship between employment and QoL are lacking (69). Therefore, it will be necessary to further explore the relationship between employment and QoL of patients with schizophrenia in the future (70–72). In this study, married participants experienced better QoL, which is consistent with the studies regarding the impact of marital status on QoL of patients with schizophrenia. In these studies, low QoL was associated with having no reliable friend or daily contact with family members, and having few leisure activities.

The present study has some study limitations. First, many factors that affect QoL, such as self-esteem and stigma, were not assessed. Second, because of a relatively low response rate at 2 years (66%). the results of this study may be more applicable to patients with stronger treatment compliance and cooperation. Third, the population of the study included patients with stable conditions treated in the community or outpatient clinics, excluding inpatients and patients in the acute phase, which could also affect the generalizability of the results. Fourth, the BPRS is not a good scale for assessing negative symptoms, especially defining the threshold for severe negative symptoms. In contrast, the PANSS scale, in relative terms, contains many more items than the BPRS for clearly defining and measuring negative symptoms (73). Therefore, future studies may need to consider using more precise scales to measure patient symptoms. Fifth, negative symptoms are divided into primary and secondary. Secondary negative symptoms can be caused by positive symptoms, depressive symptoms, and medication side-effects social deprivation substance abuse (74). However, this study did not differentiate between primary negative symptoms or secondary negative symptoms, which need to be considered in future studies. Finally, the patients enrolled in this study were 19–75 years old, spanned a large area, and did not distinguish whether the patients were first-episode or recurrent. Therefore, there is a certain impact on the generalization of the results, and future studies are warranted to further differentiate between patients with age at first presentation and recurrence.

This study has some notable strengths, including a longitudinal design and use of a large data set of standardized and validated measures to evaluate QoL in patients treated in primary mental health care within an emerging economic power - China. This study considered many factors that affect the QoL such as depression, sleep, insight, medication, and attitudes, and observed dynamically the overall QoL in patients with schizophrenia and possible influencing factors. A further strength was the statistical methodology (GEE analysis) used, which is especially suitable for repeated measures data.

## Conclusions

This study showed that QoL generally improved over the years in all domains, except the psychological QoL domain. More patients were unemployed after 2 years, used more psychotropic drugs, had more depressive symptoms, fewer negative symptoms (probably fewer secondary negative symptoms), and had worse knowledge of their illness. Further analysis found that to improve the quality of life of patients with schizophrenia in primary care, we should prioritize treatment of depressive, negative and insomnia symptoms, the choice of antipsychotic medication and improvement of the treatment compliance. But, how to operate remains a therapeutic challenge for clinicians, patients, and families because guidelines are unavailable and therapeutic interventions are poorly evaluated. More attention should probably be paid to patients with early onset of illness, older age, and unmarried patients to improve QoL.

## Data availability statement

The original contributions presented in the study are included in the article/supplementary material, further inquiries can be directed to the corresponding author.

## Ethics statement

The studies involving human participants were reviewed and approved by Public Health Ethics Committee of Guangdong Provincial People's Hospital. The patients/participants provided their written informed consent to participate in this study.

## Author contributions

All authors listed have made a substantial, direct, and intellectual contribution to the work and approved it for publication.

## Funding

This work was supported by Guangdong Provincial Foundation for Basic and Applied Basic Research Natural Science Foundation [grant number: 2022A1515010619]. This study was supported by Guangdong Provincial Innovation Platform of Translational Medicine (Early recognition and intervention of major mental diseases), and Guangdong Provincial Innovation Platform of Public Health.

## Conflict of interest

The authors declare that the research was conducted in the absence of any commercial or financial relationships that could be construed as a potential conflict of interest.

## Publisher's note

All claims expressed in this article are solely those of the authors and do not necessarily represent those of their affiliated organizations, or those of the publisher, the editors and the reviewers. Any product that may be evaluated in this article, or claim that may be made by its manufacturer, is not guaranteed or endorsed by the publisher.
